# Blood pressure surveillance in cancer patients treated with immune checkpoint inhibitors

**DOI:** 10.1038/s41371-023-00831-z

**Published:** 2023-04-19

**Authors:** Sean Tan, Ella Spear, Nikhita Sane, Adam J. Nelson, Nitesh Nerlekar, Eva Segelov, Stephen J. Nicholls

**Affiliations:** 1 Victorian Heart Institute, Monash University, Melbourne, VIC Australia; 2 Monash Heart, Monash Health, Melbourne, VIC Australia; 3 Monash University, Melbourne, VIC Australia

**Keywords:** Risk factors, Hypertension, Diagnosis

## Abstract

Immune checkpoint inhibitors (ICI) are cancer therapies that have been associated with increased risk of atherosclerotic cardiovascular disease (ASCVD). Blood pressure (BP) measurements are routinely performed during day oncology center visits for administration of ICI therapy but are often not assessed temporally to screen and monitor hypertension, which could independently increase the risk of ASCVD in cancer survivorship. This study reports the feasibility of using serial BP measurements from routine visits to day oncology center to diagnose and monitor hypertension control in cancer patients receiving ICIs.

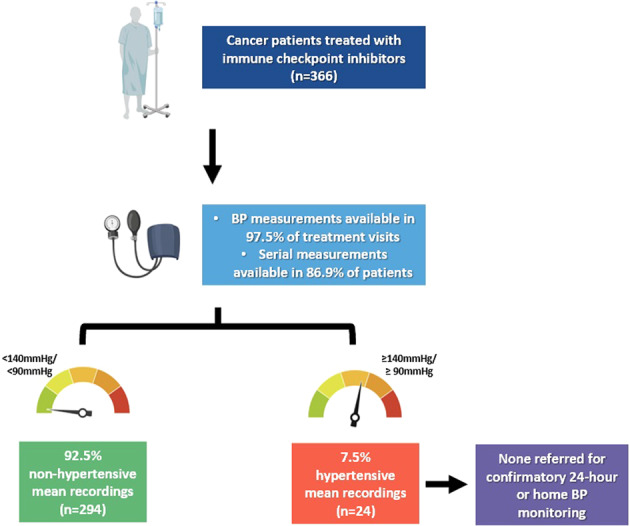

## Introduction

Immune checkpoint inhibitors (ICI) are immuno-oncological cancer therapies that have transformed the prognoses of numerous cancers [1]. However, ICIs have been associated with increased risk of long-term atherosclerotic cardiovascular disease (ASCVD) due to their inherent pro-inflammatory mechanisms [2–4]. This is further heightened by the prevalence of shared risk factors between cancer and ASCVD such as smoking, diabetes, and obesity [5]. Hence, the 2022 European Society of Cardiology (ESC) Cardio-Oncology Guidelines recommend identification and management of modifiable cardiovascular risk factors (CVRF) in patients treated with ICIs at baseline and during cancer survivorship [6]. Blood pressure (BP) measurements are routinely obtained as part of standard cancer care during hospital visits for ICI therapy to ensure clinical stability at time of ICI administration, but are often not assessed temporally for hypertension screening or surveillance. In this study, we evaluated the utility of BP surveillance among cancer patients treated with ICIs using available measurements obtained as part of standard cancer care over multiple visits for ICI administration.

We performed a single-center retrospective analysis of consecutive ambulatory patients treated with ICIs at Monash Health, Melbourne, Australia between January 2018 and December 2020. Institutional ethics was obtained for the study. Patients were identified from the hospital’s pharmacy database, using prescription data comprising available ICIs. All patients presented to a day oncology center for ICI administration at 3- or 4-weekly intervals, depending of ICI regimen. At each visit, our institutional protocol mandated BP measurements to be obtained to ensure clinical stability prior to ICI administration. Demographics, baseline CVRFs, medication history, cancer history, ICI regimen, recorded BP measurements and prescription of new antihypertensive therapies were determined from electronic medical records.

The primary endpoint was the number of patients with hypertensive readings, defined as systolic BP ≥140 mmHg or diastolic BP ≥90 mmHg. Analysis was performed using mean BP recordings among patients who had ≥2 BP measurements. Categorical variables were analyzed using Chi square analysis and continuous variables via *t*-test. A two-tailed *p* < 0.05 was considered statistically significant.

A total of 366 patients attending 3639 day oncology center visits were identified. There were 156 patients (42.6%) with documented history of hypertension prior to ICI therapy, of whom 142 (91.0%) were taking regular antihypertensives. BP measurements were documented in 3548 visits (97.5%) for ICI administration, of which 771 readings (21.7%) were hypertensive. The median number of BP measurements for the entire cohort was 6 (interquartile range 3-12). A total of 352 patients (96.2%) had baseline BP measurements prior to commencement of first ICI dose (at first day oncology center visit). The mean baseline systolic and diastolic BP was 124.0 ± 14.8 mmHg and 74.2 ± 10.4 mmHg, respectively.

There were 318 patients (86.9%) who had ≥2 recorded BP measurements available for analysis (median number of BP measurements 7 [interquartile range 4–14]). Twenty-four (7.5%) had hypertensive mean BP recordings; Of these, 12 (50%) had no known history of hypertension. Patients with hypertensive mean BP recordings had higher baseline systolic BP (142.0 ± 13.6 mmHg vs 122.6 ± 13.9 mmHg, *p* < 0.01) and diastolic BP (84.7 ± 9.9 mmHg vs 73.3 ± 10.0 mmHg, *p* < 0.01) prior to ICI initiation (Table 1). There were no differences in baseline demographics, reported CVRFs including history of hypertension, cancer history, and ICI regimen (including use of concurrent vascular endothelial growth factor inhibitor) between those with or without hypertensive mean BP recordings (Table 1). None of the patients with hypertensive mean BP recordings were documented to have confirmatory 24-h ambulatory or home BP monitoring or change in antihypertensive therapy.

**Table 1:** Baseline characteristics of patients with ≥2 blood pressure readings.

Characteristic	Non-hypertensive mean BP readings (*n* = 294)	Hypertensive mean BP readings (*n* = 24)	*p* value
Age, years	63.9 ± 11.0	68.5 ± 13.9	0.06
Male sex	181 (61.6)	17 (70.8)	0.37
Hypertension	117 (39.8)	12 (50.0)	0.33
Diabetes mellitus	47 (16.0)	5 (20.8)	0.54
Hyperlipidemia	92 (31.3)	7 (29.2)	0.83
Smoking status (current or former)	244 (83.0)	22 (91.7)	0.27
Past medical history			
Ischemic heart disease	27 (9.2)	3 (12.5)	0.59
Heart failure	7 (2.4)	0 (0.0)	0.45
Stroke	6 (2.0)	1 (4.2)	0.50
Medications			
Aspirin	46 (15.7)	5 (20.8)	0.51
Statin	92 (31.3)	5 (20.8)	0.29
RAS inhibitors	99 (33.7)	8 (33.3)	0.97
Beta blocker	33 (11.2)	4 (16.7)	0.42
Calcium channel blocker	42 (14.3)	4 (16.7)	0.75
Hydrochlorothiazide	17 (5.8)	1 (4.2)	0.74
Mineralocorticoid antagonists	3 (1.0)	1 (4.2)	0.18
Baseline BP measurements			
Systolic, mmHg	122.6 ± 13.9	142.0 ± 13.6	<0.01
Diastolic, mmHg	73.3 ± 10.0	84.7 ± 9.9	<0.01
Median number of BP measurements	7 [4–13]	7 [4–20]	0.83
Cancer Type			
Non-small cell lung cancer	196 (66.7)	18 (75.0)	0.40
Melanoma	21 (7.1)	4 (16.7)	0.10
Head and neck	27 (9.2)	1 (4.2)	0.40
Renal cell carcinoma	24 (8.2)	0 (0.0)	0.15
Small cell lung cancer	10 (3.4)	0 (0.0)	0.36
Urothelial cancer	5 (1.7)	1 (4.2)	0.39
Other	11 (3.7)	0 (0.0)	0.34
Cancer Stage			
I	3 (1.0)	0 (0.0)	0.62
II	14 (4.8)	0 (0.0)	0.27
III	105 (35.7)	9 (37.5)	0.86
IV	172 (58.5)	15 (62.5)	0.70
Prior Treatments			
Resection	59 (20.1)	5 (20.8)	0.93
Chemotherapy	223 (75.9)	16 (66.7)	0.32
Radiotherapy	145 (49.3)	11 (45.8)	0.74
VEGF inhibitor	7 (2.4)	0 (0.0)	0.45
Immune Checkpoint Inhibitor			
Ipilimumab	19 (6.5)	2 (8.3)	0.72
Nivolumab	128 (43.5)	10 (41.7)	0.86
Pembrolizumab	90 (30.6)	10 (41.7)	0.26
Durvalumab	43 (14.6)	3 (12.5)	0.78
Atezolizumab	35 (11.9)	1 (4.2)	0.25
Combination	21 (7.1)	2 (8.3)	0.83
Number of doses	11.1 ± 10.6	13.0 ± 12.8	0.40
Duration of therapy, days	197.6 ± 187.9	240.3 ± 233.2	0.29
Concurrent VEGF inhibitor	1 (0.3)	0 (0.0)	0.78

Values are mean ± standard deviation, median [interquartile range] or *n* (%).

*BP* blood pressure, *RAS* renin-angiotensin-system, *VEGF* vascular endothelial growth factor.

In this study of 366 consecutive real-world patients treated with ICIs for cancer, we have reported the feasibility of utilizing routine BP measurements obtained during standard cancer care for hypertension screening and surveillance. We found that BP measurements were obtained in 97.5% of day oncology center visits for ICI administration and serial measurements were available in 86.9% of patients. Additionally, 7.5% of patients had hypertensive mean BP readings on serial measurements, with elevated baseline readings suggesting undiagnosed or uncontrolled hypertension at time of first ICI dose. Although these results need to be interpreted with caution as patients in the study could have been in pain or anxious prior to receiving ICI therapy, these measurements did not prompt further confirmation with 24-h ambulatory or home BP monitoring or change in antihypertensive therapy. To the authors’ knowledge, this is the first study to report hypertension screening and surveillance using routine BP measurements recorded during visits to day oncology center as part of standard cancer care.

Hypertension is a prevalent modifiable CVRF, affecting approximately 32% of adults aged 40 to 59 years in the United States [7]. Although ICIs have not been associated with increased risk of developing hypertension [8], it is probable that a significant proportion of cancer patients treated with ICIs will have pre-existing or independently develop hypertension during follow up [9]. Hypertension during cancer survivorship has been shown to be associated with subsequent cardiovascular morbidity and mortality [10]. This emphasizes the growing importance for hypertension screening and control among patients treated with ICIs, especially given the improvement in cancer survivorship with ICIs and their association with ASCVD. Hence, the 2022 ESC Cardio-Oncology Guidelines recommend BP measurement every three months during the first year of ICI treatment and then six-monthly if ICI therapy persists over one year.

Regular BP measurements obtained during standard cancer care presents a pragmatic opportunity for widespread screening and monitoring of hypertension in patients treated with ICIs as recommended by guidelines. However, this is not commonly performed in routine oncological practice for a number of reasons. Firstly, BP measurements are often only assessed in isolation to ensure clinical stability prior to ICI administration. Additionally, oncologists are understandably more focused on cancer treatment and prognoses rather than BP control during standard cancer follow up, possibly leading to elevated serial BP readings being unnoticed. These challenges may be addressed by integrating automatic systems into electronic medical records to calculate mean BP readings and alert clinicians to patients who are persistently hypertensive over serial measurements. Confirmatory 24-h ambulatory or home BP monitoring can subsequently be undertaken to differentiate sustained from white-coat hypertension, thus identifying patients treated with ICIs who could benefit from lifestyle modification and subsequent antihypertensives. Streamlined referral systems to cardio-oncology services could also be adopted to allow holistic and simultaneous oncological and cardiovascular care in these patients.

The results of this study should be interpreted in the context of several limitations, including the small patient population, single-center setting, and retrospective design. Although pragmatic, it is unclear whether the BP measurements used in the study analysis were obtained in a standardized fashion according to guideline recommendations [11]. Additionally, we could not account for 24-h ambulatory BP monitoring or prescription of antihypertensive therapies that occurred outside of the single-center setting, although it would be expected these should have been documented in the patient’s medical history.

In conclusion, hypertension screening and surveillance through the repurposing of routine BP measurements obtained during standard oncological care is a feasible strategy that could be used to guide hypertension management in patients treated with ICIs. This strategy is pragmatic and could help address the increasing need for cardiovascular risk reduction during cancer survivorship as recommended by the 2022 ESC Cardio-Oncology Guidelines. This model could further be expanded to all cancer patients attending day oncology centers for administration of systemic therapies.

## Summary

### What is known about this topic

- Immune checkpoint inhibitors are associated with atherosclerotic cardiovascular disease.
- Although not associated with immune checkpoint inhibitors, hypertension is prevalent and is associated with cardiovascular mortality and morbidity in patients with cancer.
- Blood pressure is routinely checked in standard cancer care but not used to screen or monitor for hypertension.

### What this study adds

- Blood pressure measurements obtained during standard cancer care could be used to screen and monitor for hypertension.
- Cancer patients treated with immune checkpoint inhibitors may have undiagnosed hypertension.
- Patients with hypertensive readings from routine cancer care measurements could be referred for confirmatory 24-h or home blood pressure monitoring.

## Data Availability

The datasets generated during and/or analyzed during the current study are available from the corresponding author on reasonable request.
